# Developing a teaching research culture for general practice registrars in Australia: a literature review

**DOI:** 10.1186/1447-056X-8-6

**Published:** 2009-06-16

**Authors:** Marjan Kljakovic

**Affiliations:** 1 Academic Unit of General Practice and Community Health, Australian National University Medical School, Canberra, Australia

## Abstract

**Objective:**

To ascertain the issues all general practice educators need to understand when educating GP registrars to learn about research.

**Study Design:**

A review of MEDLINE [1996–2007], six websites and key informants produced 302 publications, which reduced to 35 articles, 7 books, and 9 policy documents.

**Results:**

Key themes that emerged from a thematic analysis of the literature that GP educators need to consider when teaching registrars about research were [i] the need to understand that learning research is influenced by attitudes; [ii] the need to address organisational constraints on learning research; [iii] the need to identify the educational barriers on learning research; [iv] the need to understand there are gaps in GP research content – especially from GP registrars; And [v] the need to understand the value of research on the GP registrar's educational cycle of learning, which develops in a culture that allows research to flourish.

**Conclusion:**

Australian GP registrars will observe a research culture only if they encounter clinician-researchers paid to practice and conduct research in their general practice.

## Introduction

The primary goal of most Australian general practice post-graduate training programs has been to develop a high quality clinical workforce [1]. The training programs have occurred within vocational, college, or academic contexts and their curricula have been driven by a concern to meet the workforce needs of communities, individuals, registrars and general practitioners [GPs] across Australia [1,2]. Curricula have contained an unequal amount of education and research: All curricula have concentrated on education delivered during the GP registrar-training period. None would mandate that research was to be a formal part of the curriculum. Furthermore, none would mandate that GP supervisors [or educators] develop their research teaching skills, or act as role models by undertaking research.

The relationship between education and research within general practice curricula has been shaped by culture. Medical culture can be defined as *"a way of perceiving and understanding the world by those who work in, or are served by, health care" *[3]. Cultural perspectives can be understood through an examination of language and literature used to describe the relationship between education and research. For example, the Royal Australian College of General Practitioners [RACGP] research policy statement stated that *"a commitment to research is a core aspect of general practice..." *[4]. This language of commitment placed research at the heart of any modern medical discipline, including general practice. Another example of the language of commitment were the glowing accolades given to the few GP registrars who had chosen to do research as an additional part of their general practice education [1,2,5-10].

It is not known if this cultural perspective is universal across Australian general practice, GP registrars, and their supervisors or educators. The aim of this review is to ascertain from the literature what it takes for research to become an integral part of a GP training program. The review will describe the issues all GP educators needed to consider when educating GP registrars to learn about research. The review will also report how some GP educators would develop a culture of research in the context of general practice education.

## Methods

The literature review process is shown in Table 1. A search strategy was conducted on MEDLINE 1996–2007 using combinations of key words to find references on the relationship between GP registrars, teaching, and research. The biographies of identified references were also used to expand the search. Furthermore, specific websites relevant to GP education and research in Australasia were searched. Informants were approached for suggestions including four academic GPs and Medical Educators from GP training programs in Australia and two from New Zealand.

**Table 1:** Actions taken in reviewing the literature exploring the relationship between GP registrars, GP educators, teaching and research using MEDLINE, websites, and key informants

**Literature review actions**		**Number of references**
Initial search of MEDLINE 1996–2007*		302
Two reviewers culled 302 titles and abstracts		34
Two reviewers culled 34 printed references for relevance		20
Review of the reference lists from the 20 relevant references		35
		
Review of six websites:		
PHCRED		
AGPN		7 books
AGPT		9 policy documents
RACGP		
RNZCGP		
AMAZON		
		
Discussion with key informants		0
Emerging themes were extracted by one reviewer		51

* Key words for the search strategy included *general practice *OR *family practice; general practice *OR *research; teaching *OR *education*. The term *supervisor *did not add any new references and therefore was excluded.

Two reviewers [MK, LW] independently assessed the titles and abstracts. Articles were included if they addressed the relationship between teaching and research for any type of GP educators [including GP supervisors], and registrars. Any disparity about which article to include was discussed and resolved by consensus. The concordance rate between reviewers was 0.95. A grounded theory framework informed the review [11] and the themes that emerged were extracted by one reviewer [MK].

## Results

The initial search of MEDLINE produced 302 publications, which reduced to 35 references on the teaching of research to GP registrars. Additional file 1 shows 15 references were from Australia [12-26], 12 from the USA [27-38], two from Canada [39,40] and The Netherlands [41,42], and one each from Israel [43], Taiwan [44], the United Kingdom [45], and Slovenia [46]. There were 15 surveys conducted [13,16,19,20,23,29-31,33,35-37,40,43,44]. One was a comparative survey [40], two were in-depth qualitative surveys [20,31] and two were surveys published in letter format [29,44]. There were three case studies [17,19,22], 13 editorials or commentaries [18,21,24,27,28,32,34,38,41,42,45-47], and five literature reviews published [12,14,15,26,39]. There were no experimental studies. The search of websites produced seven books [48-54] and nine policy documents [2,4-10,55] that were relevant for teaching or general practice research. None of the informants provided new references.

### Data synthesis

The themes that emerged on reviewing the 35 references, seven books, and nine policy documents are listed in Table 2 and will be explained below. There was considerable variation in the language found in the literature used to describe GPs, registrars, and educators. The list of definitions used in this review is listed in Table 3.

**Table 2:** Themes that emerged from the literature review

**A thematic analysis of 51 references found five emerging themes that GP educators needed to consider when teaching GP registrars about research:**
1. Attitudinal influences on learning research
2. Organisational constraints on learning research
3. Educational barriers on learning research
4. Gaps in the research publications by GP registrars
5. The value of research for GP registrars

**Table 3:** Definitions of individuals and organisations found in the literature review

GP	A *General Practitioner *who is a registered medical practitioner who is qualified and competent for general practice. Synonymous with *Family Practitioner *in the literature from North America.
	
GP Registrar	A GP who is undertaking training, education, and experience in general practice. Synonymous with a *GP Trainee*, or *resident *in literature from North America.
	
GP Educator	A GP employed to design and participate in a General Practice Training Program [GPTP] and takes on the role of a clinical educator. Synonymous with a *Program Director *who is usually a GP Educator who manages and directs a GPTP.
	
GP Supervisor	A GP with responsibility for registrar training in the clinical setting. Often takes on a mentorship role. Synonymous with a *GP Trainer*.
	
GPTP	*General Practice Training Program*. Synonymous with *General Practice Vocational Training Program*. An organisation providing general practice vocational training. In a number of countries, such organisations are accredited for this purpose by GP Colleges. Synonymous with *Residency Training Program *in the literature from North America.
	
Academic Staff	A person who is employed by a university to teach and do research. Synonymous with *Faculty Staff*.
	
An Academic Department	A department within a University that teaches medical students enrolled for a primary medical degree. Such departments are often involved with medical student general practice placement. In north America many *Residency Training Programs *are located within Academic Departments.
	
Student	A *medical student *enrolled with a University to gain a primary medical degree

There were six medical educators, six GPs, and seven GP registrars who published the 18 surveys and cases publications. The main emphasis of non-practice based GP educators was on resources and educational influences for teaching research to GP registrars. In contrast, GP educators and their registrars emphasised their attitudes to research, or lack of time to carry out research within clinical practice.

In the early 1990s, the literature was largely dominated by the views of medical educators and GPs about the process of teaching research. It was only later that GP registrars had their views published. The first publication by an identified GP registrar occurred in 2004 [17] and thereafter much of the GP registrar literature was published in the form of case studies. There was only one article describing the research environment for a GP registrar in an Australian general practice.

### Five Emerging themes

#### 1. Attitudinal influences on learning research

When asked, GP educators, supervisors, and registrars stated *positive feelings *to learning research [16,29-31,33-36]: Research was felt to be enjoyable to do and would advance the discipline of general practice. GP educators emphasised that academic staff should actively support research by placing it high on their program's list of priorities [16,30,31]. They should be visibly supportive of research [29] as this would set an example to encourage GP registrars to undertake research.

GPs appreciated the opportunity to participate in reputable research activities relevant to general practice and having access to information resources [16,33]. Medical Educators from the USA emphasised large research intense University Departments were important to attract more research funds and provide specific time for research for both academic staff and family medicine residents [30,31,35,36].

Negative feelings were also stated. In the Netherlands GPs struggled to find research questions that were different from hospital practice [42]. Few GP registrars endorsed the statement "doing research will make me a better clinician" and few believed that "practicing family physicians should do research" [29].

A core value in general practice is a belief that the doctor-patient relationship is important, as are the outcomes that arise from consultations [49]. As we have seen, GPs had negative feelings about teaching research. This was *feeling the difficulty *in generating good research questions and *feeling the difficulty *in being able to use complex research methods to answer questions. These powerful feelings prevent the GP from *initiating any research activity*, let alone teaching research to the GP registrar.

#### 2. Organisational constraints on learning research

Four organisational constraints on learning research were identified

##### a] A lack of finances

The fee-for-service funding environment of Australian general practice did not provide payments to offset the costs of doing research. Furthermore, organising research grant funding was difficult [16,25]. Another barrier occurred if the GP registrar needed funding to pay for a research project [29]. Some Australia Training Programs employed academic GP registrars to take part in research and many countries award scholarships [RACGP does], but only a handful had been taken up.

##### b] A lack of time

GP educators, supervisors, and registrars commented on the problem of finding time to do research within routine clinical general practice [29,30].

##### c] A lack of human resources

Research needed a critical mass of people from Academic departments and GP registrar programs [35]. A minority of residency programs in the USA made research compulsory and few programs linked annual resident promotion to progress on a research project [36]. A combination of an attitude that "research isn't necessary" plus the organisational issues of: [i] a lack of faculty staff and, [ii] a lack of time dedicated for research, has been identified as sufficient to inhibit family practice residents from undertaking research.

##### d] A lack of career enhancement

Some Australian GPs sought career enhancement through research. For example they might choose to work in universities as academics, or become GP educators working in GP training programs, but most were sceptical whether career enhancement would eventuate [25]. GP registrars could collect a boutique of specialty degrees, but this would not give added status for career enhancement in general practice. Finally, a research track record would never translate to a career enhancement in general practice – evident by the finding that Australian GPs found it difficult to use research skills in the clinical setting [25].

### 3. Educational barriers on research learning

Five educational barriers for teaching of research were identified:

a) GPs struggled to identify research questions that were different from hospital practice and relevant to general practice [42].

b) GPs struggled with research methods. Community derived research questions needed complex research methods to answer them. These methods were usually a combination of qualitative and quantitative techniques rather than the more narrow focus of quantitative methods found in hospital specialist practice [25].

c) Overly ambitious curriculum guidelines produced by various colleges that resembled a PhD curriculum [5,36].

d) Lack of recognition that a single uniform approach to research training did not exist [31].

e) Among those residents who had been in a research training program, few reported that their training prepared them to do research [29].

### 4. Gaps in research publications by GP registrars

There had been an increasing volume of published research from Australian general practice over the previous 25 years [15,26]. Table 4 lists the range of topics and suggests a number of gaps. For example, there were no research topics from rural general practice or research on health inequalities and the determinants of health in Australia. Less than half the published research focused on clinical topics [14]. There was less research published from Australian primary care if compared to the production from internal medicine, surgery, or public health [12]. This gap in publications was also found in United Kingdom [56] and The Netherlands [41] general practice.

**Table 4:** Research topics published from Australian General Practice

Research Topic	Literature Review* [1990–1999] N = 546	GPEP projects** [1990–1999] N = 501
Studies of GP behaviours, views, & opinions	28%	-
Education & Training	15%	13%
Studies of education & training	15%	-
Encounter & clinical epidemiology studies	12%	-
GP & patient behaviours	11%	-
Health service interface	9%	15%
Organisation of general practice	6%	6%
Patient behaviour, views, opinions, compliance	6%	-
Methodology	4%	12%
Workforce	3%	-
Evidence based medicine	1%	21%
Finance	1%	3%
Ethical/legal/professional	1%	3%
Service characteristics	-	24%
Quality of care	-	17%
Supply and distribution	-	5%
Health Inequalities and the determinants of health	0%	0%
Rural Health	0%	0%

*[26]**[14]

The editor of The *Lancet *argued that the presence of these gaps indicated that *"Primary care research is a lost cause" *[57]. This line has been vigorously denied by arguing that much of the clinical and preventive care that occurred in general practice must be underpinned by research produced from general practice [45]. Furthermore, the long list of common diagnoses and their management found in general practice could not be informed by hospital-based research alone [42,45].

It is a truism to state that the more common the condition in general practice, the less it is studied [58]. This truism could be applied to the GP registrar who was commonly found working throughout Australian general practice, but the largest gap in publications was seen from GP registrars. Overall, there was very little literature describing the kind of topics in which GP registrars had done research. Grzydowski described an evaluation of research projects done by 271 Canadian GPs who had been residents involved in research between 1990 and 1999. The GP residents' contribution to research consisted largely of their collecting data for projects. 40% of their projects were cross-sectional studies. Only 7% of projects were published and half the remaining residents would have liked to publish their study. There had been only two Australian case studies describing what research GP registrars did. Chien described how a GP registrar and supervisor within one general practice could do research by using an action technique for research [17]. Gartlan described her experience of being a novice researcher [19]. Montgomery published five case studies of how GP registrars produced research proposals at a research workshop [22].

### The value of research for GP registrars

There were two cultural perspectives found in the review on the value of research for the GP registrar. First, although it was widely recognised that research was part of primary care [41], many people perceived general practice as being a non-academic discipline that should concentrate mainly on the provision of health care [46]. This cultural perspective placed research in the background of what the GP registrar should learn when being taught general practice. For example, few providers of GP registrar training in Australia had included research as a compulsory project within their training [Tasmania was one exception] [1,59]. Research had also played a minor role in Academic Family Practice in the USA [36].

A consequence of the low value placed on research was that the GP registrar became a late author of research in the history of general practice research. The first publication by a GP registrar in this review was in 2004 [17]. The first Australian GP training programs started over two decades before that time.

The second perspective found in the review placed research into the foreground of general practice by arguing the benefits of research. These benefits included an increase in the status of general practice in academia, improved professional standards, and improved standardisation of terminology and diagnostic and therapeutic procedures [41]. GPs could obtain personal benefit from research in that it improved morale, reduced dissatisfaction to working in general practice and improved intellectual stimulation [41]. The benefits could also be extended to the GP registrar. In addition, the GP registrar could gain educational benefits from performing research tasks including asking questions, critical appraisal and creating new knowledge. Such benefits were at the centre of the educational cycle of learning [11].

Svab argued that a culture of research would exist if clinician-researchers were paid to do general practice and conduct research at the same time, and also worked with a critical mass of interdisciplinary researchers [46]. Svab suggested that a cultural change was required to develop "practices of excellence' throughout a country where clinician-researchers worked within supportive organisational frameworks. A profound change in cultural mind-set was required. GPs needed to change from not only being the traditional devoted and caring doctor of individual patients, but also a GP who became research oriented. If a GP registrar were to stumble upon "a practice of excellence", he or she would find a research culture where all individuals within the practice had a *way of perceiving and understanding the world that included research*.

### Stories of the GP registrar doing research in general practice

This review found a few successful stories of learning research in general practice: GPs were more likely to undertake research if they had prior research experience in their undergraduate, medical school, or residency period [33]. Individual GPs told success stories of how they started research as a young GP and went on to built a life of research [14]. There is an account of a registrar and a GP supervisor working together in a practice environment where a research culture is clearly evident [19].

Stories about a research culture have as yet to be articulated in general practice. The literature suggests such stories would contain five elements. The first would include the active involvement of research mentors [16,21,32,33,35]. Such mentors would be found through colleges, university departments or research networks. They would be medical educators, supervisors, faculty staff, or GPs, actively involved in research. The second element would include a supportive teaching infrastructure for research [21,29,31,33]. This structure would provide administrative resources, organise dedicated time and funding. The third element would require GP registrars to create and publish small research projects [21,36]. The final element would include a description of people who have an inherent enjoyment of research [33].

### Enabling factors increasing research participation by registrars

The review identified six enabling factors that not only increased research participation by registrars but also changed the negative cultural perspective. First, there should be an increase in the number of GP registrars who undertake research. The more registrars completing a project during training, the more likely it was that they would do research in their career [35]. Second, more GP registrars should observe clinician-researchers doing their work in general practice [34]. Third, many more GP registrars should become research fellows [34]. Fourth, a GP Training Program should be linked to a collaborative research network in order to develop a critical mass of researchers [34,46]. Fifth, a GP Training Program should provide all local GP educators and supervisors access to information resources and opportunities to participate in relevant research activities [16]. Finally, GP educators should start talking about general practice research early in a medical student's career pathway. Medical students should asked questions relevant to general practice when they engage in their learning about general practice [31,42].

## Discussion

The review found that the GP registrar was a late arrival in the history of general practice research. The literature was dominated largely by the views of medical educators and GPs about the process of teaching research.

A thematic analysis of the literature revealed the need for GP educators or supervisors to understand that learning research was influenced by attitudes. They needed to address organisational influences, accept that research influenced the educational cycle of learning, and understand there are gaps in what GP registrars actually did in research. Research among Australian GP registrars will flourish if they find themselves working in a research culture. This will occur only when they encounter clinician-researchers paid to practice and conduct research in their daily clinical practices.

Achieving a research culture in Australian general practice will require work. A change in the current research culture in Australia requires GP educators and supervisors to develop their our own research agendas, collaborate widely with other GP researchers in the world, and develop an Australian-based criteria for success [46]. One such change would be to place research at the centre of the educational cycle of learning [11]. The GP registrar is better educated when developing the art of asking questions and answering them. The GP registrar learns by gaining new knowledge through this cycle and applying the new knowledge to the care of patients in general practice. An ability to ask questions and answer them is also important for medical science. This art is best developed when doing research.

## Competing interests

The author declares that they have no competing interests.

## About the author

Marjan Kljakovic MB CHB, FRNZCGP, PhD, Professor of General Practice Academic Unit of General Practice & Community Health, Australian National University Medical School, Canberra, ACT

*Correspondence*: marjan.kljakovic@anu.edu.au

## Supplementary Material

Additional file 1 **Summary of Literature on GPs, GP registrars, GP supervisors, teaching, and research between 1996 and 2007**. Additional table Click here for file
