# Supplementary material for: Developing a teaching research culture for general practice registrars in Australia: a literature review
Source: Asia Pac Fam Med. 2009 Jun 16;8(1):6. doi: 10.1186/1447-056X-8-6 (PMC2706233; doi:10.1186/1447-056X-8-6)
Supplement: Additional file 1 — Summary of Literature on GPs, GP registrars, GP supervisors, teaching, and research between 1996 and 2007. Additional table [file 1447-056X-8-6-S1.doc]

**Additional File**

**TABLE Summary of Literature on GPs, GP registrars, GP supervisors, teaching, and research between 1996 and 2007**

| **Author** | **Reference** | **Country** | **Year** | **Method** | **Response**  **[% = Percent]** | **Themes** |
| --- | --- | --- | --- | --- | --- | --- |
| Hueston | (33) | USA | 1996 | Survey | N= 55  [74%] | Described the enabling and inhibiting features for GPs and residents in family residency programs. Sample frame was GPs who had published at least one article |
| Clarke | (18) | Australia | 1996 | Editorial | - | Describes the research process |
| Giveon | (43) | Israel | 1997 | Survey | N= 190  [94%] | Described the factors that dictated involvement in research |
| DeHaven | (30) | USA | 1997 | Survey | N= 321  [75%] | Described the enabling and inhibiting features for Family residency programs |
| Oeffinger | (37) | USA | 1997 | Survey | N=373 programs directors  N= 112 family medicine chairs  [75%] | Described the enabling and inhibiting features for Family residency programs. |
| Costa | (29) | USA | 1998 | Letter | - | Described the enabling and inhibiting features for residency programs [in response to de Haven] |
| DeHaven | (31) | USA | 1998 | Qualitative survey | N= 28 | Characteristics of program success from GP program directors & registrars [purposeful selection of 28 successful Training Programs] |
| Grzybowski | (39) | Canada | 1999 | Review | N= 251 | Described the type of projects done by residents from 1990-1997 |
| Rodnick | (38) | USA | 1999 | Commentary | - | Commentary of Research Fellowships available in USA for residents to develop their research skills. |
| Ward | (26) | Australia | 2000 | Review | Medline Review of 20 years of literature | Medline Review of 20 years of literature |

| **Author** | **Reference** | **Country** | **Year** | **Method** | **Response**  **[% = Percent]** | **Themes** |
| --- | --- | --- | --- | --- | --- | --- |
| Mainous | (35) | USA | 2000 | Survey | N= 124  [55%] | Department chairs described the enabling and inhibiting features for research-intense academic departments of Family Medicine [top 40 for NIH funding] & less intense Departments |
| Kaczorowski | (40) | Canada | 2001 | Comparative study | 70% for research stream versus 66% for normal curriculum | Described the comparison. No particular benefit in having a research stream on whole school. But did foster research among those who did the stream |
| Fortin | (32) | USA | 2001 | Commentary | - | Commented on what would it take to encourage residents to do research |
| Shah | (24) | Australia | 2001 | Commentary | - | Commentary of GP research in Australia |
| Askew | (12) | Australia | 2001 | Review | - | Compared GP research output with other disciplines |
| Dinant | (42) | Netherlands | 2002 | Editorial | - | Describes the research process from the GP perspective |
| Neale | (36) | USA | 2002 | Survey | N= 416  [75%] | Program directors described the enabling and inhibiting features for residency programs |
| Askew | (13) | Australia | 2002 | Survey | N= 476  [76%] | Described GP attitudes to research |
| Beacham | (15) | Australia | 2003 | Review | N= 52 projects | A range of common topics described arising from general practice. GPEP projects 1999-2001 |
| Mainous | (34) | USA | 2003 | Commentary | - | Described the research process needed for GPs to increase their publication output. |
| Carek | (27) | USA | 2003 | Editorial | - | Reviewed Hueston and Mainous on being successful with family medicine residency research. |

| **Author** | **Reference** | **Country** | **Year** | **Method** | **Response**  **[% = Percent]** | **Themes** |
| --- | --- | --- | --- | --- | --- | --- |
| Lee | (44) | Taiwan | 2003 | Letter | N= 143 | Faculty members described how neither time given for research by the department, nor amount of time in ambulatory care determined research activity. |
| van Weel | (41) | Netherlands | 2004 | Commentary | - | Building GP research capacity globally |
| Mant | (45) | UK | 2004 | Commentary | - | A defence of research in primary care |
| Chien | (17) | Australia | 2004 | Case study | N= 2 | Shows how a GP/ GP trainee pair is able to work together through research. |
| Hiramanek | (21) | Australia | 2004 | Commentary | - | Describes the research process of undertaking a small project in the general practice |
| Carek | (47) | USA | 2004 | Editorial | - | Promoting scholarly activity in family medicine residency programs |
| Svab | (46) | Slovenia | 2004 | Commentary | - | Changing a research culture |
| Gartlan | (19) | Australia | 2005 | Case study | - | Describes how a GP trainee did research |
| Trevena | (25) | Australia | 2005 | Qualitative project | N=16 | GPs gave their opinions of the facilitators and barriers to research. GPs with prior research training show an interest in research,. |
| Carek | (28) | USA | 2005 | Editorial | - | Listing strategies for promoting scholarly activity in family medicine residency programs |
| Government | (14) | Australia | 2005 | Review | - | Overview of GP research in Australia |
| Beilby | (16) | Australia | 2003 | Survey | N = 463  [84%] | GPs describe their attitudes and enabling factors for research [Cluster of unrepresentative sample of GPs from division] |
| Gartlan | (59) | Australia | 2006 | Survey | N= 40  [90%] | GP supervisors describe their attitude to research |
| Reid | (23) | Australia | 2006 | Survey | N= 121  [60%] | GPs and GP registrars described the impact of a research workshop weekend on registrars doing research |
| Montgomery | (22) | Australia | 2007 | Case series | N= 5 | GP registrars present the kind of research projects they have done. The cases were used in a research workshop |

| **Document type** | **Year** | **Author** | **Title** |
| --- | --- | --- | --- |
| **General Practice Research books** | 1982  1989  1990  1991  1992  1997  1998 | Stephens  Howie  Armstrong  Norton  Stewart  McWhinney  Frey | The Intellectual Basis of Family Practice (52)  Research in General Practice (49)  Research Methods for General Practitioners (48)  Primary Care Research (51)  Tools of Primary Care Research (53)  A Textbook of Family Medicine (50)  The Clinical Philosophy of Family Medicine (54) |
| **Policies** | 2003  2003  2004  2005  2005  2006  2006  2006  2006 | Dickinson  Olesen  Dickinson  RACGP  RNZCGP  AAFP  CFPC  RCGP  Snowden | Research and the Society of Teachers of Family Medicine (7)  A framework for clinical general practice and for research and teaching (8)  STFM responsibilities for AFMO Research Strategic Planning the discipline (6)  Standards for General Practice Education and Training. Program & Providers (2)  RNZCGP Strategic Plan(10)  AAFP Research Policy (5)  Research & Education Foundation (55)  RCGP Research Curriculum (9)  RACGP Research Policy(4) |
